# Increased Risk of Cerebrovascular Events in Patients with Cancer Treated with Bevacizumab: A Meta-Analysis

**DOI:** 10.1371/journal.pone.0102484

**Published:** 2014-07-15

**Authors:** Pei-Yuan Zuo, Xing-Lin Chen, Yu-Wei Liu, Chang-Liang Xiao, Cheng-Yun Liu

**Affiliations:** Key Laboratory of Geriatrics of Health Ministry, Department of Geriatrics, Union Hospital, Tongji Medical College, Huazhong University of Science and Technology, Wuhan, China; University of North Carolina School of Medicine, United States of America

## Abstract

Arterial ischemia and hemorrhage are associated with bevacizumab, an inhibitor of vascular endothelial growth factor that is widely used to treat many types of cancers. As specific types of arterial ischemia and hemorrhage, cerebrovascular events such as central nervous system (CNS) ischemic events and CNS hemorrhage are serious adverse events. However, increased cerebrovascular events have not been uniformly reported by previous studies. New randomized controlled trials (RCTs) have been reported in recent years and we therefore conducted an up-to-date meta-analysis of RCTs to fully characterize the risk of cerebrovascular events with bevacizumab. We searched the databases of PubMed, Web of Science, and the American Society of Clinical Oncology conferences to identify relevant clinical trials up to February 2014. Eligible studies included prospective RCTs that directly compared patients with cancer treated with and without bevacizumab. A total of 12,917 patients from 17 RCTs were included in our analysis. Patients treated with bevacizumab had a significantly increased risk of cerebrovascular events compared with patients treated with control medication, with a relative risk of 3.28 (95% CI, 1.97–5.48). The risks of CNS ischemic events and CNS hemorrhage were increased compared with control, with RRs of 3.22 (95% CI, 1.71–6.07) and 3.09 (95% CI, 1.36–6.99), respectively. Risk varied with the bevacizumab dose, with RRs of 3.97 (95% CI, 2.15–7.36) and 1.96 (95% CI, 0.76–5.06) at 5 and 2.5 mg/kg/week, respectively. Higher risks were observed in patients with metastatic colorectal cancer (RR, 6.42; 95% CI, 1.76–35.57), and no significant risk was observed in other types of tumors. In conclusion, the addition of bevacizumab significantly increased the risk of cerebrovascular events compared with controls, including CNS ischemic events and CNS hemorrhage. The risk may vary with bevacizumab dose and tumor type.

## Introduction

The overexpression of vascular endothelial growth factor (VEGF) has been observed in several tumor types and is associated with a poorer patient prognosis [1]. VEGF binds to and activates a receptor tyrosine kinase, stimulating the growth of blood vessels, which plays a central role in the growth, invasion and metastasis of tumors. Disruption of VEGF signaling is a major focus of new cancer therapeutics. Bevacizumab, a humanized recombinant monoclonal antibody against VEGF, was first authorized in the USA in 2004 for the treatment of metastatic colon and rectal cancer. To date, bevacizumab has been approved by the US Food and Drug Administration for the treatment of metastatic colorectal cancer (mCRC), advanced non-squamous non-small-cell lung cancer (NSCLC), glioblastoma and metastatic renal cell carcinoma (mRCC).

Bevacizumab has been shown to increase the risk of arterial ischemia and serious hemorrhage [2], [3], [4]. However, there is no evidence supporting an association with increased CNS ischemic events or CNS hemorrhage, the specific types of arterial ischemia and hemorrhage. Ranpura et al. conducted a meta-analysis in 2010 and found that bevacizumab increased the risk of cardiac ischemia; however, the risk of ischemic stroke with bevacizumab was not significantly different from that of controls [5]. Likewise, in 2010, Hapani et al. reported that the risk of CNS hemorrhage with bevacizumab appeared to be low [3]. Carden et al. concluded that no trial reported evidence supporting an increased risk of intracranial bleeding during anti-VEGF therapy, even in the presence of CNS metastases [6]. Cerebrovascular events are adverse events leading to morbidity and mortality in patients with malignancy, and although infrequent, they are life threatening. CNS bleeding was reported to be the cause of death in one-third of patients who experienced a bleed [7]. Therefore, it is imperative to find out whether such cerebrovascular disorders develop as a result of bevacizumab treatment.

New RCTs have been performed during the past three years [8], [9], [10], [11], [12]. Although not significantly different when compared with controls, several studies have reported a higher incidence of CNS ischemia or CNS hemorrhage with bevacizumab [8], [9], [10]. We consider that individual trials may be limited in patient number and that the previous meta-analyses were not sufficiently large to reveal a significantly increased risk of cerebrovascular events in patients with bevacizumab. To further understand these issues, we conducted an up-to-date, thorough literature search and meta-analysis to characterize the impact of bevacizumab on the occurrence of cerebrovascular events in cancer patients.

## Methods

### Data Source

We performed a comprehensive search of citations from PubMed between January 1966 and February 2014, using the keywords “bevacizumab”, “avastin”, and “carcinoma/cancer”. The search was limited to randomized clinical trials. We also searched abstracts and virtual meeting presentations from the American Society of Clinical Oncology conferences held between January 2004 and November 2013, using the keywords “bevacizumab” or “avastin” and “randomized”. An independent search using the citation database Web of Science was also conducted to ensure that no clinical trials were missed. The search was limited initially to English publications in humans. We screened the reference lists of the included studies and related publications. The results were then hand searched for eligible trials. The results were double-checked and arbitrated by a second investigator.

### Study Selection

The purpose of this study was to determine whether bevacizumab contributes to the development of CNS ischemic events or CNS hemorrhage in patients with cancer. Therefore, we selected for analysis only those randomized clinical trials that directly compared patients with cancer treated with and without bevacizumab. Trials that met the following criteria were included: (1) prospective phase II and III randomized clinical trials in patients with cancer, (2) randomized assignment of participants to bevacizumab treatment or control in addition to current chemotherapy and/or biological agent, (3) direct comparison of patients with and without bevacizumab therapy with data available for CNS ischemic events or CNS hemorrhage.

### Data Extraction and Quality Assessment

Data abstraction was conducted independently by two investigators (P.Y.Z. and Y.W.L.) to avoid bias in the data-abstraction process, and any discrepancies between reviewers were resolved by consensus. For each study, we extracted the following information: first author’s name, year of publication, trial phase, underlying malignancy, number of enrolled patients, treatment arms, median follow-up and bevacizumab dose. CNS ischemic events and CNS hemorrhage were defined and assessed according to the National Cancer Institute’s common terminology criteria for adverse events (version 1, 2 or 3), which has been widely used in cancer clinical trials (Additional file, Table S1) [13]. Version 1 was used in 1 trial [14]; version 2 was used in 3 trials [15], [16], [17]; version 3 was used in 12 trials [8], [9], [10], [11], [12], [18], [19], [20], [21], [22], [23], [24], and the one of the trials did not specify [25]. Minor variation exists among these versions in grading CNS ischemic events and CNS hemorrhage. Data regarding the occurrence of cerebrovascular events were obtained from the safety profile of each study. Criteria were assessed for quality including randomization and allocation concealment, blinding, sample size, exclusions after randomization, and different lengths of follow-up [26].

### Statistical Analysis

STATA 12.0 (STATA Corp, College Station, Texas) and RevMan 5 (http://ims.cochrance.org/revman/download) were used for statistical analysis. For the calculation of incidence, the number of patients with cerebrovascular events and the number of patients treated with bevacizumab were extracted from the safety profiles of the selected clinical trials. The proportion of patients with cerebrovascular events and the 95% CIs were derived from each trial. We also calculated the RRs and CIs of cerebrovascular events in patients assigned to bevacizumab versus controls. To explore a dose–effect relationship, bevacizumab therapy was further divided into low dose (2.5 mg/kg per week) and high dose (5 mg/kg per week). The designation of low vs. high dose is relatively arbitrary. We also conducted subgroup analyses type of cerebrovascular events and underlying malignancy.

Statistical heterogeneity among trials included in the meta-analysis was quantified with the *I* ^2^ statistic (100%*[Q-*df*]/Q), which estimates the percentage of total variation across studies due to heterogeneity rather than chance [27]. If the *I* ^2^ value was greater than 50%, the assumption of homogeneity was deemed invalid, and the random-effects model was reported after exploring the causes of heterogeneity. Otherwise, the fixed-effects model was reported [28]. Publication bias was evaluated with Begg’s and Egger’s tests [29], [30]. A two-tailed P-value<0.05 was considered statistically significant.

## Results

### Population Characteristics and Quality

**Figure 1:**
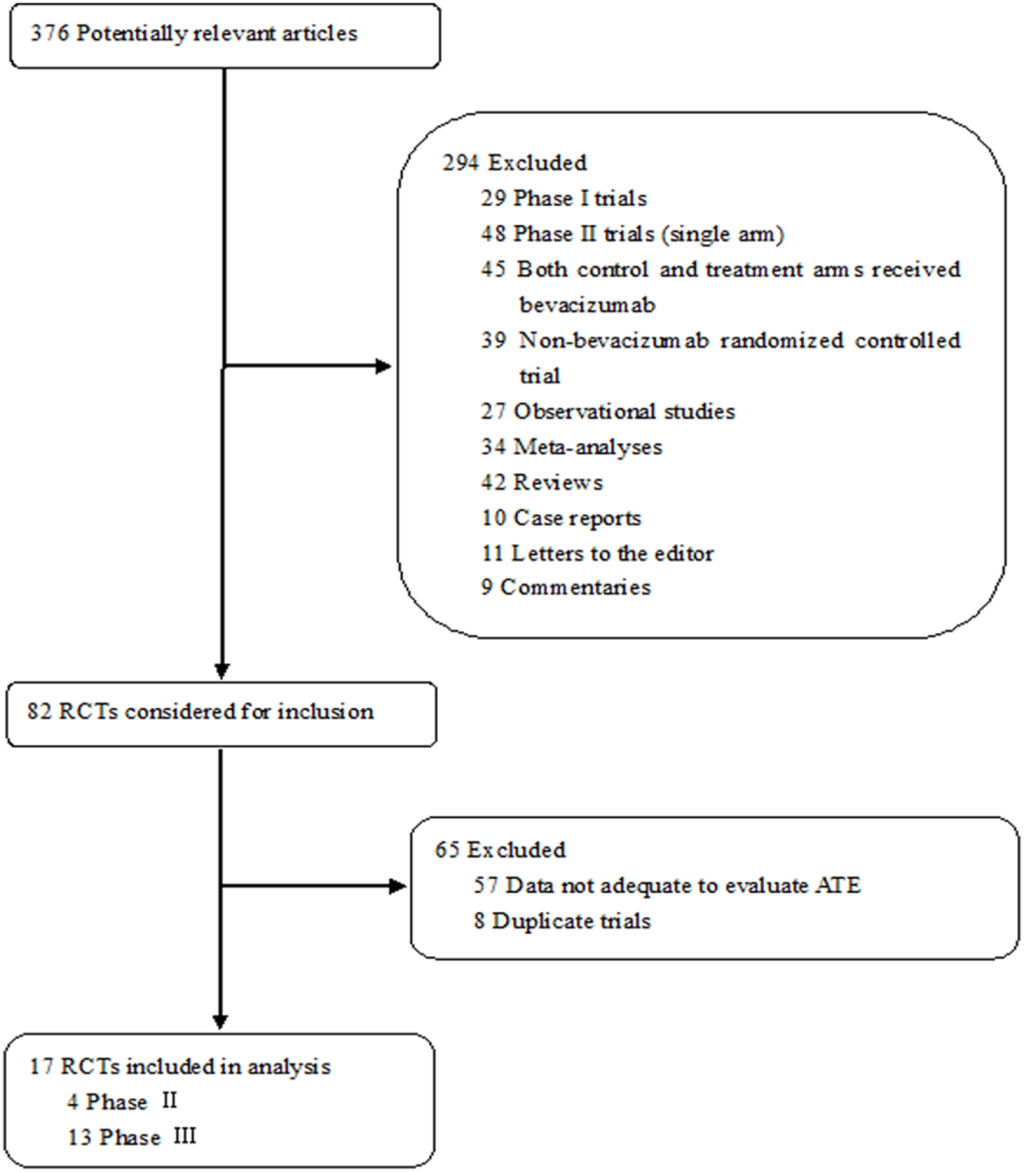
Selection process for the randomized clinical trials included in the meta-analysis.

Our initial search yielded a total of 376 clinical studies relevant to bevacizumab. After excluding review articles, phase I, single-arm phase II studies, case reports, meta-analyses, observational studies, RCTs with both arms containing bevacizumab, and data not adequate to evaluate cerebrovascular events, we selected 17 randomized controlled trials. The detailed selection process is represented in Figure 1.

The characteristics of each RCT are presented in Table 1. The baseline Eastern Cooperative Oncology Group performance status for most patients was between 0 and 1. Patients were required to have adequate organ function, coagulation and hematologic parameters. Patients were excluded if they had significant cardiovascular disease, uncontrolled hypertension, serious non-healing wounds, major surgery within the previous 28 days, bleeding diathesis or significant coagulopathy, brain metastasis, or known CNS disease; if they required parenteral hydration or nutrition; or if they were receiving specific drugs thought to increase the risk of bleeding.

**Table 1:** Characteristics of randomised controlled clinical trials included in the meta-analysis.

Firstauthor,year	Trialphase	Patientsenrolled	Patients foranalysis	Medianfollow-up(months)	Underlyingmalignancy	Concurrenttreatment	Reportedcerebrovascular events	Bevacizumabdose(mg/kg Per week)^a^
Aghajanian, [12] 2012	III	543	480	24	recurrent ovarian, primary peritoneal, or fallopian tube cancer	Gemcitabine and carboplatin	CNS hemorrhage	5
Allegra, [19] 2009	III	2710	2647	28.5	Colorectal cancer	Fluorouracil, leucovorin, and oxaliplatin	CNS ischemia	2.5
Burger, [24] 2011	III	1873	1209	17.4	Ovarian cancer	Paclitaxel and carboplatin	CNS bleeding	5
Chinot, [8] 2014	III	921	921	NA	Glioblastoma	Radiotherapy, temozolomide	CNS bleeding	5
Giantonio, [17] 2007	III	829	572	28	Colorectal cancer	Oxaliplatin, fluorouracil, and Leucovorin	Cerebrovascular ischemia/hemorrhage	5
Herbst, [22] 2011	III	636	626	19	Non-small-cell lung cancer	Erlotinib	CNS hemorrhage	5
Kabbinavar, [14] 2003	II	104	102	17.6.	Colorectal cancer	Fluorouracil and leucovorin	Cerebrovascular accident	5
Kelly, [9] 2012	III	1050	1009	25	Prostatic cancer	Docetaxel and prednisone	Cerebrovascular ischemia	5
Kim, [23] 2012	II	214	212	13	Melanoma	Carboplatin and paclitaxel	CNS hemorrhage	5
Kindler, [18] 2007	II	115	108	15.6	Mesothelioma	Gemcitabine and cisplatin	Cerebrovascular accident	5
Kindler, [21] 2010	III	602	540	NA	Pancreatic cancer	Gemcitabine	Cerebrovascular accident	5
Miller, [16] 2007	III	722	711	41.6	Breast cancer	Paclitaxel	Cerebrovascular ischemia	5
Okines, [11] 2013	II	213	200	22	Gastro-oesophageal adenocarcinoma	Epirubicin, cisplatin and capecitabine	Cerebrovascular thromboembolic event	2.5
Perren, [25] 2011	III	1528	1528	NA	Ovarian cancer	Paclitaxel and carboplatin	CNS bleeding	2.5
Price, [10] 2012	III	471	471	30.8	Colorectal cancer	Capecitabine and Mitomycin	Cerebrovascular ischemia	2.5
Rini, [20] 2010	III	732	709	NA	Renal cell carcinoma	Interferon alfa	Cerebrovascular ischemia	5
Sandler, [15] 2006	III	878	867	19	Non-small-cell lung cancer	Paclitaxel and carboplatin	CNS hemorrhage/accident	5

Abbreviations and notes: NA, data not available; CNS, central nervous system.

Founding sources: three trials were sponsored by Genentech [12], [14], [23], eleven trials were supported by National Cancer Institute and National Institute of Health [9], [15]–[22], [24], [25], One trial was supported by Roche Australia [10], One trial was supported by Cancer Research UK [11], One trial was supported by F. Hoffmann–La Roche [8].

a The dose schedule was converted from mg/kg per schedule.

We did not differentiate between high- and all-grade CNS ischemic events or CNS hemorrhage, as very few studies reported on all-grade or low-grade (grade 1–2) CNS ischemia or bleeding. All the RCTs reported CNS ischemic events as high (grade 3–5). One RCT reported all-grade CNS bleeding [12], and one RCT did not mention any grade [24], all other RCTs reported CNS bleeding as high grade.

In all trials, patients were randomly assigned to either a control or bevacizumab group, with two three–arm studies each having two bevacizumab-treatment groups [10], [17], in which patients received different combinations. Eight trials were placebo-controlled, double-blinded studies [8], [9], [12], [18], [21], [22], [23], [24], and the rest of the trials had active controls [10], [11], [14], [15], [16], [17], [19], [20], [25]. The quality of all the trials was acceptable.

### Risk of Cerebrovascular Events

A total of 12,917 patients from 17 RCTs were identified and included for analysis. Among the patients who were administered bevacizumab, the meta-analysis revealed that the summary incidence of cerebrovascular events was 0.5% (95% CI, 0.3%–0.7%), as shown in table 2.

**Table 2:** Incidence and RR of cerebrovascular events with bevacizumab among patients with various tumor types.

Subgroup	No. ofstudies	CVE no./total noBevacizumab	Control	Incidence(95% CI), %	RR (95% CI), P
Overall	16	59/6421	14/6284	0.5 (0.3–0.7)	3.28 (1.97–5.48), <0.00001
Colorectal cancer	4	14/1960	5/1877	0.5 (0.2–0.7)	2.28 (0.90–5.73), 0.37
Metastatic colorectal cancer	3	9/634	0/556	0.7 (0.1–1.2)	6.42 (1.16–35.57), 0.03
NSCLC	2	6/740	0/753	0.3 (−0.1–0.7)	7.14 (0.88–57.60), 0.54
Ovarian cancer	3	6/1600	1/1587	0.2 (0–0.3)	3.42 (0.72–16.35), 0.84
Others	7	33/2121	8/2067	0.9 (0.6–0.12)	3.59 (1.75–7.38), 0.0005

NSCLC, non-small-cell lung cancer; CVE, cerebrovascular events; CI, confidence interval; RR, relative risk.

The observed incidences of cerebrovascular events with bevacizumab may have been influenced by other confounding factors, such as concurrent chemotherapy, underlying malignancy, hypertension, diabetes, hyperlipidemia, and medications. To assess the particular contribution of bevacizumab to the risk of cerebrovascular events and to exclude the influence of confounding factors, we determined the overall RR of cerebrovascular events from the RCTs in which patients were treated with or without bevacizumab in combination with concurrent standard antineoplastic therapy.

**Figure 2:**
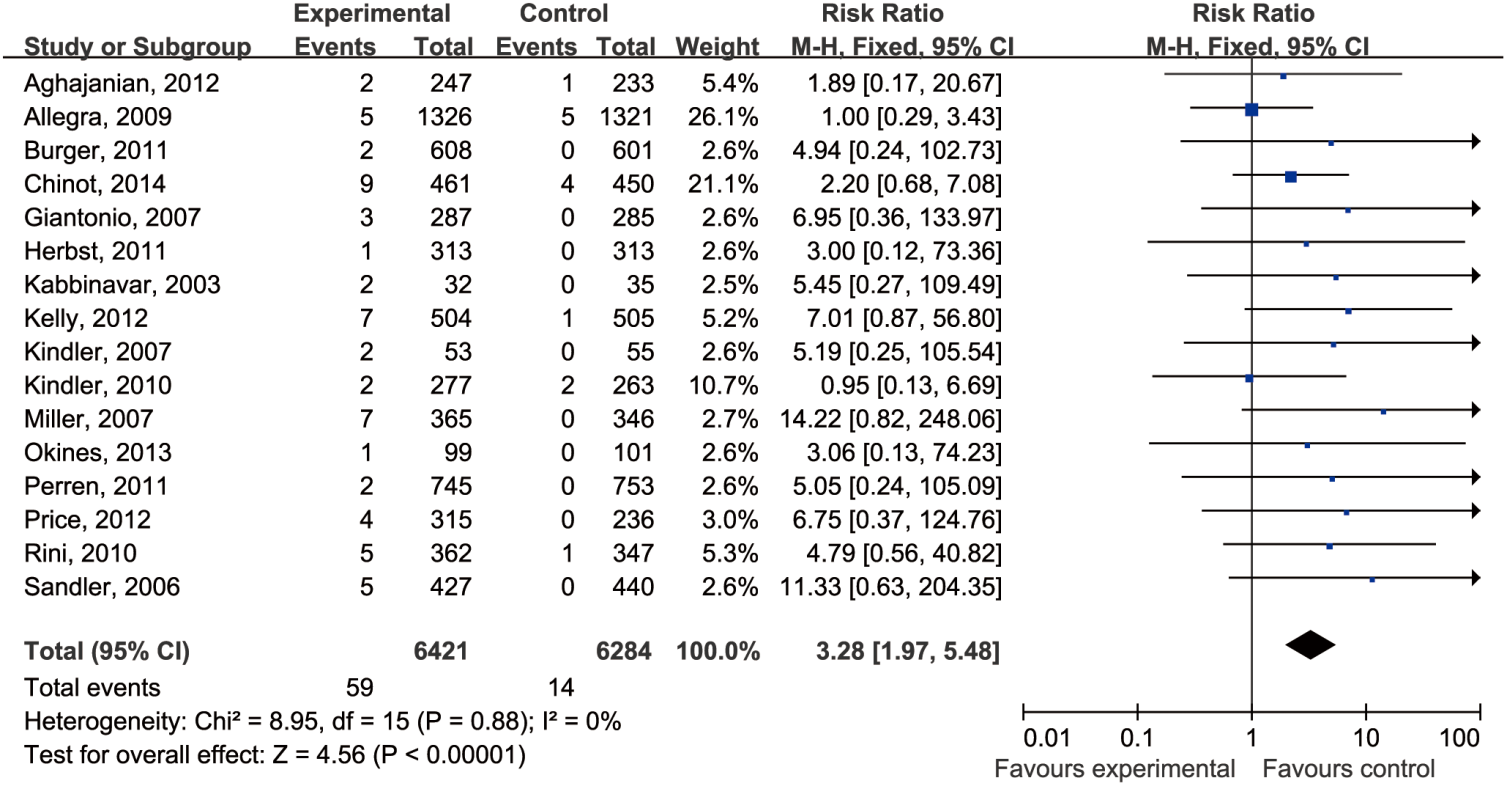
Relative risk (RR) of cerebrovascular events associated with bevacizumab versus control. The overall RR of cerebrovascular events was calculated using a fixed-effects model. The areas of the squares are proportional to the weights used for combining the data. The diamond plot represents the overall results of the included trials. One trial was not included in the final calculation of relative risk [23] because neither the control nor the bevacizumab groups had events.

We performed a meta-analysis of 16 RCTs (one trial was not included in the final calculation of relative risk because neither the control nor bevacizumab groups had cerebrovascular events). No heterogeneity was found among the studies included in the analysis despite clear disparity in tumor type and related treatment (Figure 2). Using a fixed-effects model, the summary overall RR for bevacizumab versus control was 3.28 (95% CI, 1.97–5.48). Thus, bevacizumab was found to increase the risk of cerebrovascular events significantly.

### Risk of CNS Ischemic Events and CNS Hemorrhage

Among the patients who were administered bevacizumab, meta-analysis revealed that the incidence of CNS ischemic events was 0.5% (95% CI, 0.3%–0.7%), with the highest incidence (1.9%) observed in the malignant mesothelioma trial [18] and the lowest incidence (0.2%) found in a colorectal cancer trial [17] (Table 2). The incidence of CNS hemorrhage was 0.3% (95% CI, 0.1%–0.5%), with the highest incidence (1.4%) observed in a glioblastoma trial [8] and the lowest incidence (0.1%) found in an ovarian cancer trial [25].

**Figure 3:**
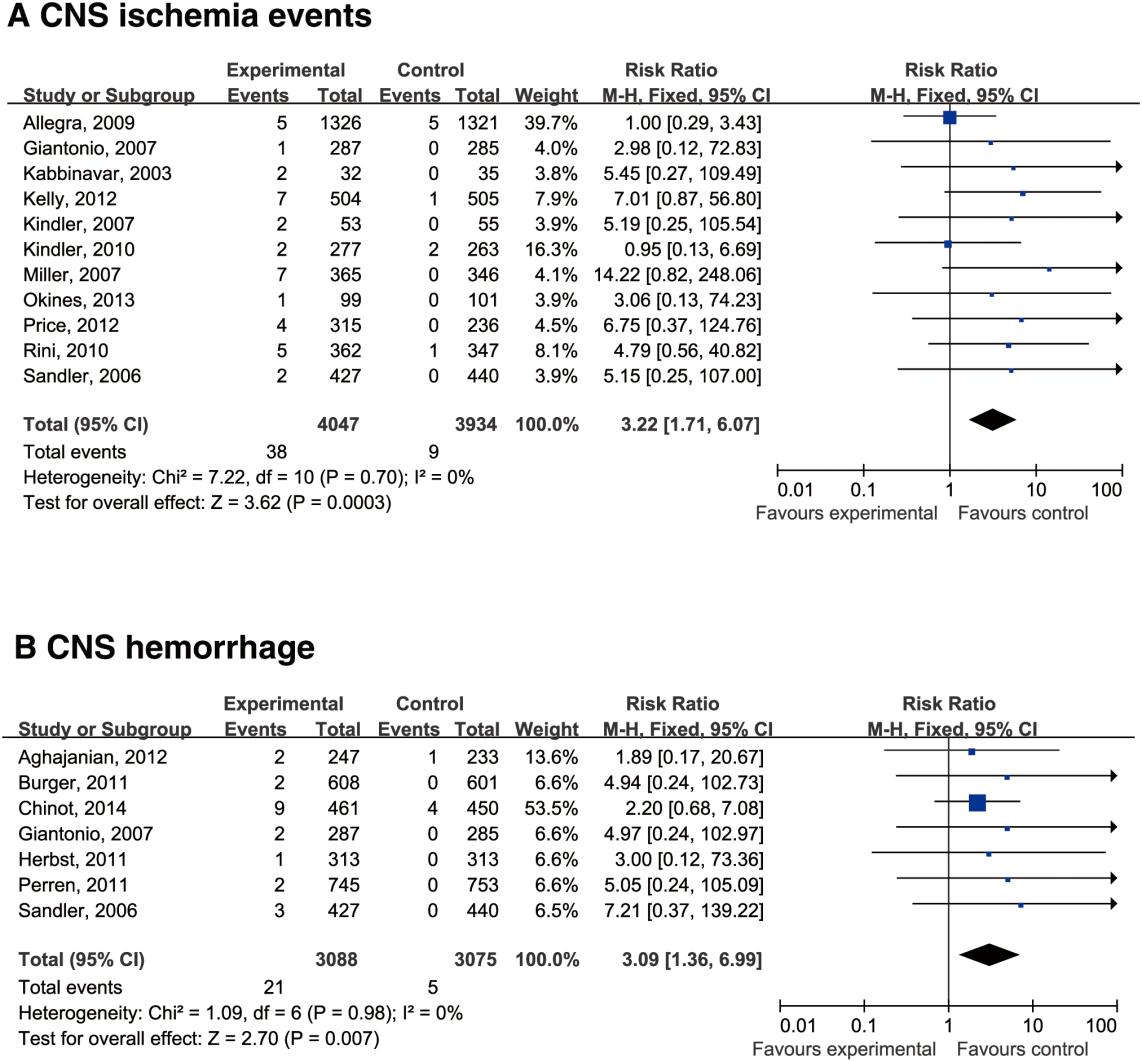
Relative risk (RR) of CNS ischemic events and CNS hemorrhage associated with bevacizumab versus controls. The RRs of CNS ischemic events (A) and CNS hemorrhage (B) were calculated using a fixed-effects model. The areas of the squares are proportional to the weights used for combining the data. The diamond plot represents the overall results of the included trials.

Using a fixed-effects model, the RR of CNS ischemic events was determined to be 3.22 (95% CI: 1.71–6.07) in comparison with controls (Figure 3). The RR of CNS hemorrhage was determined to be 3.09 (95% CI: 1.36–6.99) in comparison with controls. Therefore, the RRs of CNS ischemic events and CNS hemorrhage in patients receiving bevacizumab were 222% and 209% greater than control treatment, respectively. This finding suggested that bevacizumab significantly increased the risk of CNS ischemic events and CNS hemorrhage.

### Risk of Cerebrovascular Events and Tumor Type

Patients with different tumors might be at different risks of cerebrovascular events due to differences in tumor biology and associated treatments. As an exploratory analysis, stratified analysis was performed based on the underlying malignancy. The highest incidence of cerebrovascular events was found in patients with malignant mesothelioma (1.9%, 95% CI 0.7–4.4), while the lowest incidence was observed in patients with ovarian cancer (0.1%, 95% CI 0.1–0.3).

Notably, the relative risk of cerebrovascular events was 6.42 (95% CI, 1.76–35.57) for metastatic colorectal cancer treated with bevacizumab compared with control (Table 2).

### Cerebrovascular Events and Bevacizumab Dose

**Figure 4:**
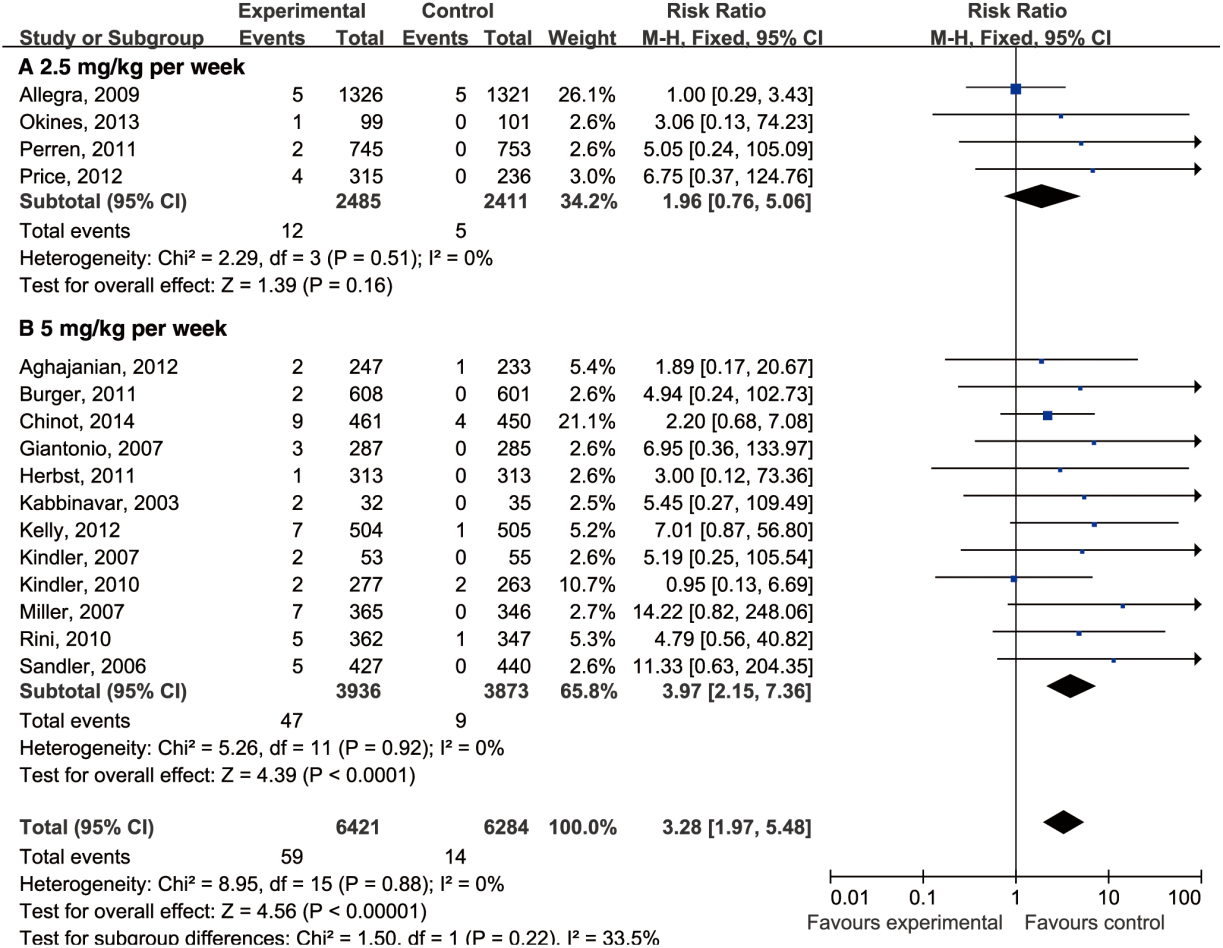
Relative risk (RR) of cerebrovascular events associated with bevacizumab at 2.5 or 5 mg/kg/week when compared to controls. Summary of the RRs of cerebrovascular events for patients receiving bevacizumab at 2·5 mg/kg per week (A) or 5 mg/kg per week (B), which were calculated using a fixed-effects model. The areas of the squares are proportional to the weights used for combining the data. The diamond plot represents the overall results of the included trials.

We assessed whether the dose of bevacizumab is related to the risk of cerebrovascular events. A meta-analysis was performed to calculate the RR associated with bevacizumab at 2.5 or 5 mg/kg/week when compared with controls. The RR of cerebrovascular events for bevacizumab at 2.5 mg/kg/week was 1.96 (95% CI, 0.76–5.06) when using four RCTs including 4896 patients. The RR of cerebrovascular events at 5 mg/kg/week was 3.97 (95% CI, 2.15–7.36) when using 12 RCTs including 7809 patients. This finding suggests that the risk of cerebrovascular events with bevacizumab was dose dependent (figure 4).

### Publication Bias

We used Begg’s and Egger’s tests to determine the presence of publication bias regarding our primary end point (RR of cerebrovascular events). The two-tailed p-values were 0.452 and 0.187 for Begg’s and Egger’s tests, respectively. Thus, no publication bias was detected.

## Discussion

Most previous studies have focused on the development of arterial thromboembolic events, such as pulmonary embolism and myocardial infarction [31], following bevacizumab treatment. Limited information is available on the effects of bevacizumab on the cerebral vasculature [32]. Our study demonstrated that bevacizumab was associated with a significantly increased risk of cerebrovascular events (RR, 3.28; 95% CI, 1.97–5.48), including CNS ischemic events (RR, 3.22; 95% CI, 1.71–6.07) and CNS hemorrhage (RR, 3.09; 95% CI, 1.36–6.99). The clinical significance of cerebrovascular events in cancer patients is evident because of their association with significant morbidity and mortality. As bevacizumab is used extensively in routine cancer treatment and clinical trials, it is important to improve the diagnosis and management of the cerebrovascular side effects of bevacizumab. This study may help physicians and patients to properly understand the risk of cerebrovascular events in bevacizumab therapy.

Mechanisms were involved in the predisposition to thrombosis and bleeding after the inhibition of VEGF signaling. VEGF not only promotes endothelial cell proliferation but also stimulates endothelial cell survival. Inhibition of VEGF could thereby impair the endothelial cells regenerative capacity, leading to thrombosis or hemorrhage [33]. VEGF also plays a role in vascular protection, with effects on endothelial cells mediated through its anti-apoptotic, anti-inflammatory and pro-survival effects. Bevacizumab may increase the expression of proinflammatory cytokines, damaging blood vessels in the brain. VEGF increases the production of NO and prostacyclin and suppresses pathways involved in endothelial cell activation, apoptosis, and coagulation [34]. Reductions in NO and prostacyclin may predispose patients to thromboembolic events. In addition, bevacizumab significantly increases the risk of hypertension in cancer patients [35], [36]. The incidence of all-grade hypertension in patients receiving bevacizumab is 23.6%, and sustained hypertension permanently changes the architecture of the blood vessels, making them narrow, stiff, deformed and uneven and therefore more vulnerable to fluctuations in blood pressure. High blood pressure greatly contributes to CNS hemorrhage.

At present, controversy remains as to whether Bevacizumab increases the risk of cerebrovascular events. The association of bevacizumab with increased risks of CNS ischemic events and CNS hemorrhage may have been underestimated by previous meta-analyses. Ranpura et al. included five RCTs reporting ischemic stroke from 2003 to 2010 and demonstrated that the RR of developing stroke with bevacizumab was 1.37 (95% CI: 0.67–2.79) [5]. Carden et al. demonstrated that even in the presence of known brain metastases, anti-VEGF therapy appears to be safe, with no recorded episodes of intracerebral hemorrhage [6]. In a meta-analysis conducted by Hapani et al. including two RCTs in 2010, the RR of CNS hemorrhage with bevacizumab was 6.01 (95% CI 0.72–50.0) [3]. We considered that the failure to detect the increased risk with bevacizumab in CNS ischemic events and CNS hemorrhage was likely due to the limited number of clinical trials included in these subgroup analyses.

New RCTs have been performed during the past several years. We collected a total of 17 RCTs reporting cerebrovascular events. Eleven of these RCTs reported CNS ischemic events, and eight reported CNS hemorrhage. Our analysis demonstrated that the risks of CNS ischemic events and CNS hemorrhage were increased over three-fold in patients treated with bevacizumab compared with controls. Importantly, the risk of cerebrovascular events in patients with CNS metastases receiving bevacizumab was not fully evaluated. Evidence is lacking in the metastatic setting because patients with brain metastases have largely been excluded from phase II and phase III clinical trials.

We explored the relationship between bevacizumab dose and cerebrovascular events. We showed that high-dose bevacizumab (5 mg/kg/week) significantly increased the risk of cerebrovascular events, with an RR of 3.26 (95% CI, 1.81–5.86) in comparison with controls, whereas low-dose bevacizumab (2.5 mg/kg/week) did not, with an RR of 1.71 (95% CI, 0.62–4.70). This result suggests that the increased risk of bevacizumab-associated cerebrovascular events is dose dependent.

This study also showed that the risk of cerebrovascular events with bevacizumab can vary with tumor type. The risk was significantly increased in patients with metastatic colorectal cancer who received bevacizumab (RR, 6.42; 95% CI, 1·16–35.57). This result is consistent with that addressed in a cohort study in patient with stage IV colorectal cancer published in 2012 [37]. The higher risk of cerebrovascular events observed in patients with metastatic colorectal cancer might be associated with the adjuvant therapies that the patients received, which have known cerebrovascular side effects [14], [17]. Another possible explanation is that the VEGF level was highest in patients with mCRC in comparison with other cancers [38]. No significant risk was found for other tumor types. In patients with glioblastoma, the incidence of CNS hemorrhage was high (1.4%; 95% CI, 0.7%–2.2%) [8]. Surgery or radiotherapy may contribute to this high incidence in glioblastoma patients [39]. To establish definitively that bevacizumab increases the risk of cerebrovascular events in tumor types except mCRC, further large-scale RCTs are needed.

Phase II or III RCTs have tight inclusion and exclusion criteria. Patients were excluded if they do not have adequate organ function, coagulation and hematologic parameters. Therefore, the RCTs from our report rarely included patients with baseline significant uncontrolled hypertension or a prior history of cerebrovascular events. It is conceivable that the rate of cerebrovascular events could be even higher in a real-life with underlying vascular comorbidities [2]. For patients receiving bevacizumab, physicians should be highly vigilant for any signs of cerebrovascular disorders. If a cerebrovascular event is detected, prompt assessment and treatment are warranted.

Our study had several limitations. First, because of the number of RCTs in each tumor type was limited, we did not determine the exact tumor type associated with significant risk. Several tumor types only included one RCT, such as prostate cancer, mesothelioma, pancreatic cancer, breast cancer and renal cell carcinoma. Further RCTs are needed. Second, all the studies were conducted at various institutions, and the ability to detect cerebrovascular events might vary among these institutions, which could result in a potential bias of recording adverse events. The incidence of cerebrovascular events showed significant heterogeneity among the included studies. Calculation using the random-effects model for incidence estimation may minimize this issue. The RRs reported by all of the studies were remarkably non-heterogeneous. Third, this was a meta- analysis conducted at the study level, and confounding factors and specific risk factors at the patient level could therefore not be assessed and incorporated into the analysis. Substantial variation exists among these trials due to patient selection (localized disease in the Allegra trial and metastatic disease in other trials), tumor type, trial phase, and concurrent treatment. Finally, there could be a potential observation time bias because bevacizumab is often associated with prolonged progression-free survival.

In conclusion, our study indicates that the novel anti-angiogenic agent bevacizumab is associated with a significantly increased risk of cerebrovascular events, including both CNS ischemic events and CNS hemorrhage, in patients with cancer receiving concurrent chemotherapy. The risk might vary with tumor type, with higher risks observed in patients with metastatic colorectal cancer. The risk was increased in patients receiving higher doses of bevacizumab. Future studies are recommended to investigate risk reduction.

## Supporting Information

Table S1 **National Cancer Institute’s common terminology criteria versions 1–3 for cerebrovascular events.**
(DOC)

Checklist S1 **PRISMA 2009 Checklist.**
(DOC)
