# Supplementary material for: Increased Risk of Cerebrovascular Events in Patients with Cancer Treated with Bevacizumab: A Meta-Analysis
Source: PLoS One. 2014 Jul 15;9(7):e102484. doi: 10.1371/journal.pone.0102484 (PMC4099178; doi:10.1371/journal.pone.0102484)
Supplement: Table S1 — National Cancer Institute’s common terminology criteria versions 1–3 for cerebrovascular events. (DOC) [file pone.0102484.s001.doc]

Table S1. National Cancer Institute’s common terminology criteria versions 1-3 for cerebrovascular events

| Grade | Version 1 | Version 2 | Version 3 |
| --- | --- | --- | --- |
| 1 | **Ischemia:** none | **Ischemia:** none | **Ischemia:** none |
|  | **Hemorrhage:** none | **Hemorrhage:** none | **Hemorrhage:** Asymptomatic, radiographic findings only |
| 2 | **Ischemia:** none | **Ischemia:** none | **Ischemia:** Asymptomatic, radiographic findings only |
|  | **Hemorrhage:** none | **Hemorrhage:** none | **Hemorrhage:** Medical intervention indicated |
| 3 | **Ischemia:** none | **Ischemia:** transient ischemic event or attack (TIA) | **Ischemia:** transient ischemic event or attack (TIA) |
|  | **Hemorrhage:** none | **Hemorrhage:** bleeding noted on CT or other scan with no clinical consequences | **Hemorrhage:** Ventriculostomy, ICP monitoring, operative intervention indicated |
| 4 | **Ischemia:** none | **Ischemia:** permanent event (e.g., cerebral vascular accident) | **Ischemia:** Cerebral vascular accident (CVA, stroke), neurologic deficit >24 hrs |
|  | **Hemorrhage:** none | **Hemorrhage:** hemorrhagic stroke or hemorrhagic vascular event (CVA) with neurologic signs and symptoms | **Hemorrhage:** Life-threatening consequences; neurologic deficit or Disability |
| 5 | none | Death | Death |
